# Breaking the Magic: Mouth and Genital Ulcers with Inflamed Cartilage Syndrome

**DOI:** 10.7759/cureus.1743

**Published:** 2017-10-04

**Authors:** Stella Pak, Shaina Logemann, Christine Dee, Adam Fershko

**Affiliations:** 1 Internal Medicine, Kettering Medical Center; 2 Wright State University Boonshoft School of Medicine

**Keywords:** magic syndrome, behcet’s disease, relapsing polychondritis

## Abstract

Mouth and genital ulcers with inflamed cartilage (MAGIC) syndrome refers to a condition in which features of Behcet’s disease (BD) and relapsing polychondritis (RP) occur in the same individual. The existence of MAGIC syndrome suggests a potential common etiology for BD and RP. However, connecting these two diseases and referring to this condition as MAGIC syndrome might have been premature, as there is currently insufficient knowledge on BD and RP. In this critical review, we argue that these two clinical entities could possibly be unique disease processes rather than two ends of the same disease spectrum. Distinguishing the clinical difference between BD and RP is critical for the management of patients diagnosed with MAGIC syndrome, as biological therapeutic approaches for BD and RP differ. Also, inaccurate perception regarding the relationship of these two diseases could mislead researchers in their endeavors to unravel the pathophysiological mechanisms behind these two diseases.

## Introduction and background

Behcet’s disease (BD) is a chronic relapsing multisystem disease characterized by inflammatory ulcers in the mouth, genital area, and skin [1]. Due to the systemic nature of BD, many experts speculate that BD can be linked to other diseases such as Crohn’s disease, Hughes-Stovin syndrome and Sweet syndrome [2-4]. Relapsing polychondritis (RP) is autoimmune recurrent chondritis of the ears, nose, larynx, and tracheobronchial tree [5]. Clinicians have been intrigued by a possible connection between BD and RP. This theory led to the establishment of mouth and genital ulcers with inflamed cartilage (MAGIC) syndrome, which was first described by Firestein in 1985 as a syndrome consisting of recurrent oral and genital ulcers and cartilage inflammation of the ears, nose, throat, and rib cage. The diagnosis of MAGIC syndrome essentially synthesized the symptoms of BD and RP into one clinical entity [6]. However, the accumulating body of knowledge regarding BD and RP points away from the perception of the two diseases as one fluid spectrum. It is possible that cartilage inflammation observed in BD could merely be a complication of BD. As both diseases are relapsing, episodic manifestations of BD involving cartilage could be mistaken as RP.

In this critical review, we argue that these two clinical entities could possibly be unique disease processes rather than two ends of the same disease spectrum. Distinguishing the clinical difference between BD and RP is critical for the management of patients diagnosed with MAGIC syndrome, as biological therapeutic approaches for BD and RP differ. Also, inaccurate perceptions regarding the relationship of these two diseases could mislead researchers in their endeavors to unravel the pathophysiological mechanisms behind these two diseases.

## Review

Difference in epidemiology

BD usually presents in the third or fourth decades of life and is most prevalent in Turkey, Japan, Korea, and Middle-Eastern and Mediterranean countries. In general, BD does not exhibit any gender predilection [1]. In contrast, RP has been reported to occur mostly in the fifth decade of life with a female predominance—RP occurs 2.5 to 3 times more often in females than in males. However, gender predisposition of RP is controversial, as several studies did not show a difference among genders regarding the incidence of RP [5]. No familial or geographical clustering of RP has been reported.

Difference in diagnostic criteria

BD is diagnosed clinically. Although 17 different sets of diagnostic criteria have been developed, the most widely utilized criteria is the International Study Group (ISG) criteria. ISG criteria require the presence of oral aphthosis in addition to two of the following symptoms: genital aphthosis, skin manifestations, ophthalmological manifestations, and positive pathergy test [7].

Diagnosis of RP is made mostly on clinical grounds based on the Michet diagnostic criteria. Major criteria are 1) inflammation in the auricular cartilage, 2) inflammation in the nasal cartilage, and 3) inflammation in the laryngotracheal cartilage. Minor criteria are 1) ocular involvement as manifested by conjunctivitis, episcleritis, scleritis, or uveitis, 2) hearing loss, 3) vestibular dysfunction, and 4) seronegative polyarthritis. For a patient to be diagnosed with RP, he or she must meet either two major criteria or one major criterion plus two minor criteria [8].

Difference in clinical presentation

BD and RP share intriguing similarities in clinical presentation, which likely led to the connection between these two diseases. BD often manifests by oral and genital aphthae, uveitis, arthritis, epididymitis, and skin lesions resembling erythema nodosum, palpable purpura, and pyoderma gangrenosum. Also, patients with BD exhibit pathergy phenomenon [9]. In contrast, auricular and nasal chondrite are most common symptoms in patients with RP. Arthritis, inspiratory dyspnea, and ocular manifestations, such as scleritis, episcleritis, and uveitis, also frequently occur in this patient population [10]. RP is frequently associated with myelodysplasia, whereas no association between BD and myelodysplasia has been reported [11].

Difference in cardiovascular manifestations

BD involves the cardiovascular system in approximately 30% of patients and can manifest in many ways, including pericarditis, coronary aneurysm or stenosis, intra-cardiac thrombus, and mitral or aortic valve prolapse/insufficiency [12]. Notably, aneurysms can affect the abdominal aorta in BD, although the carotid or pulmonary arteries have been affected per reports. Aneurysms on carotid or pulmonary arteries also have been repeatedly reported in this patient population. Similarly, RP’s most common cardiovascular involvements are aortic or mitral prolapse and insufficiency, pericarditis, and aortic aneurysm; however, aneurysms occur most frequently at the aortic root rather than the abdominal aorta [13-14].

Inflammatory aneurysms in both BD and RP involve prominent lymphocytic inflammation in the vasa vasorum [15]. However, in BD, lymphocytes tend to spare the internal elastic lamina, mostly targeting the medial and adventitial layers, while in RP, lymphocytes mostly target intima and media [16].

Difference in pathophysiology

Both diseases are hypothesized to involve genetic predisposition plus a trigger, which interact to produce the disease process. People with HLA-B51/B5 are more likely to develop autoimmune dysfunction that gives rise to various symptoms in BD. Some theorize that possessing the HLA-B51/B5 gene constitutes genetic predisposition, and the trigger is Streptococcus or herpes virus type-1 [17]. Presence of HLA-DR4 is known as a risk factor for development of RP. The disease activity of RP is positively correlated with the serum level of interferon-γ, interleukin-2, interleukin-12, suggesting a Th1-mediated process. The most strongly agreed upon hypothesis for RP disease process is that collagen type II and/or matrilin-1 act as the autoantigens triggering autoimmune inflammation [18].

Difference in treatment

The therapeutic goals for both BD and RP are symptomatic management and immunosuppression. The overlapping therapeutic modalities for both diseases are as follows: 1) colchicine 2) corticosteroids 3) immunosuppressants, namely methotrexate, azathioprine, cyclophosphamide, and 4) biological therapy, with infliximab being the most commonly used biological agent [19-20]. However, some therapeutic agents demonstrate different efficacy in BD and RP. Interferon-α and thalidomide are commonly used to treat BD; however, these are rarely used in patients with RP due to low efficacy [19, 21]. Dapsone is a common agent used in RP treatment, but it is not typically used to treat BD [20].

## Conclusions

BD and RP have many overlapping characteristics, such as autoimmune dysfunction, relapsing inflammatory episodes, systemic involvement, and rarity. However, there are also significantly different features, such as genetic background, distribution of vascular involvement and mechanism of aneurysm development. By identifying differing features of BD and RP, we challenge the view of BD and RP as different ends on the spectrum of the same disease process.

MAGIC syndrome, which is thought to be an intermediate condition between RP and BD, could possibly be cartilaginous inflammation representing systemic involvement of BD. The earlier ulcer presentation in MAGIC syndrome tends to support the view that MAGIC syndrome is truly the development of polychondritis secondary to relapsing episodes of BD. Furthermore, polychondritis is associated with autoimmune systemic diseases. Nonetheless, when evaluating the current knowledge, firm conclusions cannot be drawn about the association between BD and RP. As the etiological issues continue to be debated, the possible relationship between the two conditions has major practical implications in diagnosis and treatment. Although the approach to treating both dieases is similar in aiming to control symptoms and achieve immunosupression, different immunosuppressive agents appear to have effectiveness in each diease. Thus, distinguishing BD from RP in MAGIC syndrome patients is imperative in order to initiate nuanced treatment for the underlying disease.
